# Ferroptosis in cardiovascular diseases: role and mechanism

**DOI:** 10.1186/s13578-023-01169-2

**Published:** 2023-12-15

**Authors:** Qi Zhang, Yuhao Luo, Lin Peng, Xi Rong, Yingxue Liu, Jiafu Li, Jing Luo

**Affiliations:** 1 Department of Cardiology, The Affiliated Hospital of Southwest Medical University, Luzhou, China; 2 Department of Oncology, The Affiliated Hospital of Southwest Medical University, Luzhou, China; 3 Department of Bone and Joint Surgery, The Affiliated Hospital of Southwest Medical University, Luzhou, China; 4 Collaborative Innovation Centre for Prevention and Treatment of Cardiovascular Disease of Sichuan Province, Southwest Medical University, Luzhou, China

**Keywords:** Regulated cell death, Ferroptosis, Cardiovascular disease, Corona Virus-2

## Abstract

In multicellular organisms, regulatory cell death is a crucial aspect of growth and development. Ferroptosis, which was postulated roughly ten years ago, is a mode of cell death that differs from apoptosis, autophagy, and pyrodeath. This distinct pattern of cell death is triggered by an imbalance between oxidants and antioxidants and strongly associated with the metabolism of iron, lipids, amino acids, and glutathione. A growing body of research has implicated ferroptosis in the incidence and progression of many organ traumas and degenerative diseases. Recently, ferroptosis has gained attention as a crucial regulatory mechanism underlying the initiation and development of a variety of cardiovascular diseases, including myocardial ischemia/reperfusion injury, cardiomyopathy, arrhythmia, chemotherapy, and Corona Virus-2-induced cardiac injury. Pharmacological therapies that inhibit ferroptosis have great potential for the management of cardiovascular disorders. This review discusses the prevalence and regulatory mechanisms of ferroptosis, effect of ferroptosis on the immune system, significance of ferroptosis in cardiovascular diseases, and potential therapeutic value of regulating ferroptosis in a variety of heart diseases.

## Background

Recent studies have linked various types of controlled cell death to cardiovascular diseases (CVDs) [1–4]. Ferroptosis is a recently discovered iron-dependent regulated cell death mode that is distinguished from other regulated cell death modes by the accumulation of lipid hydroperoxides and the distinct mechanism [5, 6].

Iron-dependent accumulation of reactive oxygen species (ROS) and consumption of polyunsaturated fatty acids (PUFAs) in biofilms are the mechanisms underlying ferroptosis, which is initiated by inhibitors of glutathione biosynthesis or the glutathione-dependent antioxidant enzyme glutathione peroxidase 4 (GPX4). When intracellular lipid ROS levels exceed the antioxidant activity of GPX4, redox homeostasis is broken, which eventually leads to cell death [7]. However, sex hormones can reduce iron decline and ultimately inhibit iron death by modulating MBOAT1 and MBOAT2 expression to reshape cellular phospholipid profiles [8].

CVDs have a significant impact on public health and are the leading cause of death and disability worldwide. A multinational study showed that the CVD burden is increasingly trending towards younger age groups [9]. Moreover, recent studies have found associations between ferroptosis and a variety of CVDs, including ischemia/reperfusion injury (I/R) [10], heart failure (HF) [11], cardiomyopathy [12], and atherosclerosis (AS) [13]. For example, AS involves the toxic accumulation of lipids in vessel walls, and in AS-related cells, such as macrophages, vascular smooth muscle cells, and endothelial cells, GPX levels are low, iron metabolism is dysregulated, and ROS are elevated [13, 14].

In this review, we discuss research on the prevalence and regulatory mechanisms of ferroptosis, the effect of ferroptosis on the immune system, and the significance of ferroptosis in cardiovascular diseases. Finally, we evaluate current therapies for CVD that target ferroptosis to reveal their challenges and prospects.

## Overview of ferroptosis

Cell death is a crucial aspect of healthy biological development; however, abnormal cell death is implicated in a wide range of diseases [15]. For a long time, cell death was considered uncontrolled; however, in the 1950s, the concept of “programmed cell death” was suggested and adopted. Today, the concept of “regulated cell death,” which also refers to pyroptosis and necroptosis, is well accepted [16, 17].

Ferroptosis was identified as a new type of cell death since 2012, and it can be prevented by the antioxidant ferrostatin-1 [18]. In addition, different types of cell death connected to iron and oxidative stress have been discussed for years [19]. The connection between glutamate- and cysteine-induced cytotoxicity and cancer cell death may have initially inspired the concept of ferroptosis [20, 21], and research on oxidative stress-induced non-apoptotic cell death in neurons was reported in 2001 [22]. Erastin was reported to cause cancer cells to perish via undetermined mechanisms in 2003 [23]. A number of substances were later identified as having the ability to cause an iron-dependent cell death pattern with features distinct from those of known cell death modes [18, 24, 25]. The term ferroptosis was coined by Dixon et al. in 2012 for this type of iron-dependent cell death [18].

## Regulation of ferroptosis

**Fig. 1:**
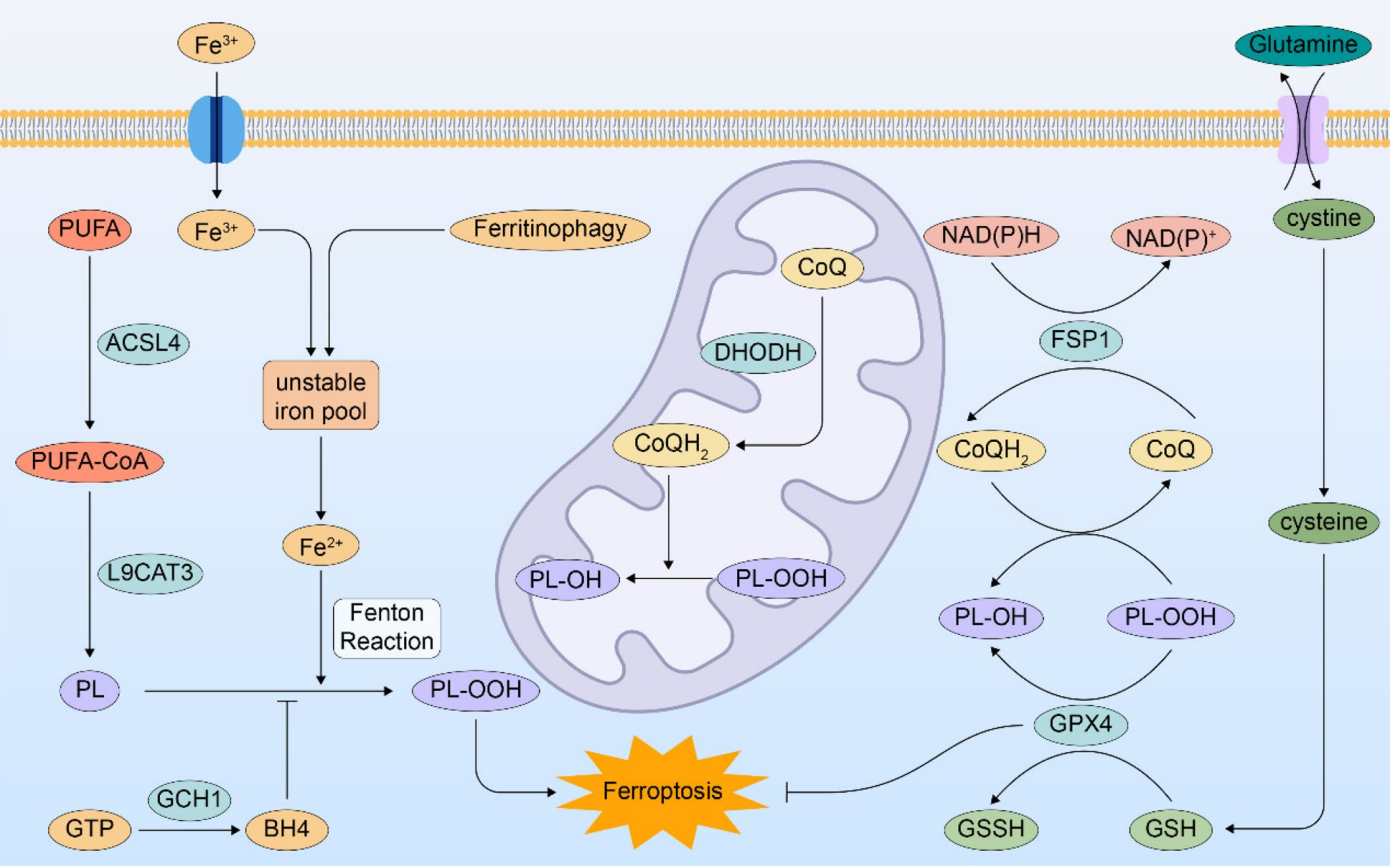
Regulatory mechanism of ferroptosis. The unstable iron pool oxidizes the PUFA on the cell membrane through the Fenton reaction and eventually leads to ferroptosis. GPX4 reduces PL-OOH to PL-OH in the presence of GSH and thus inhibits ferroptosis. DHODH in mitochondria and FSP1 reduce PL-OOH to PL-OH by providing CoQH2. GTP directly inhibits PUFA oxidation by increasing BH4

Iron, lipid, and amino acid metabolism governs the ferroptosis process, which is triggered when REDOX imbalance manifests as uncontrolled lipid peroxidation. GPX4 and a variety of antioxidant systems independent of GPX4 regulate ferroptosis. Here, we generalize the oxidation mechanisms and antioxidant mechanisms of ferroptosis (Fig. 1).

### Oxidation mechanisms

#### Lipid peroxidation

Unsaturated fatty acids play a major role in cell membrane lipid peroxidation, which is driven by free radicals, and its products increase during ferroptosis. PUFA peroxidation is crucial for ferroptosis despite the fact that a variety of cell membrane lipids may undergo oxidation [26, 27].

Long-chain-fatty-acid-coenzyme A (CoA) ligase 4 (ACSL4) and lysophosphatidylcholine acyltransferase 3 (LPCAT3) are enzymes that can incorporate PUFAs into the cell membrane. Either an enzymatic catalyst or a non-enzymatic free radical chain reaction can be used to achieve PUFA oxidation [28, 29]. The two major PUFAs that cause ferroptosis are arachidonic acid (AA) and adrenic acid (AdA) [26]. ACSL4, for example, catalyzes CoA to link with AA to create CoA-AA intermediates, which are esterified to phosphatidyl ethanolamine by LPCAT3, resulting in arachidonic acid-phosphatidyl ethanolamine (PE-AA). Both types of PE-AA oxidation, enzymatic (by lipoxygenase) and non-enzymatic (by autooxidation to PE-AA-OOH), eventually result in cell death [18, 30–32]. The ability of ACSL4 to effectively link CoA with long-chain PUFAs, such as AA and AdA, allows it to play a part in the process of ferroptosis. These long-chain PUFAs can then be re-esterified in phospholipids by different LPCAT enzymes. Short-chain monounsaturated fatty acyl tails (MUFAs) replace long-chain PUFA tails in phospholipids because of the genetic deletion of ACSL4 [26, 28]. Exogenous MUFA administration, MUFA synthesis upregulation, and ACSL3-dependent MUFA membrane accumulation can all decrease the likelihood of ferroptosis [30, 33, 34]. Therefore, one of the main methods of desensitizing cells to ferroptosis may be inhibiting ACSL4 expression, which is controlled by a number of signalling pathways [35, 36].Conversely, increased ACSL4 expression may contribute to ferroptosis in various pathophysiological settings [37, 38].

#### Iron metabolism

The Fenton reaction, for which iron is crucial, catalyses the peroxidation of PUFA-phospholipids (PUFA-PLs) through non-enzymatic automatic oxidation [39, 40]. Ferritin autophagy increases susceptibility to ferroptosis by increasing unstable iron pools through ferritin degradation [41–43]. Ferritin is recruited by nuclear receptor coactivator 4 (NCOA4) to selective cargo receptors on autophagosomes [44]. Targeting NCOA4 may be an important protocol for regulating unstable iron pools and controlling susceptibility to ferroptosis. The generation of ROS by iron-dependent Fenton reactions and the activation of iron-containing enzymes that regulate lipid peroxidation and redox homeostasis, such as arachidonate 5-lipoxygenase (ALOX), are two methods by which excess iron controls lipid peroxidation [30, 45]. In doxorubicin-induced mouse cardiomyopathy, an iron-deficient diet mitigated doxorubicin-induced myocardial toxicity and led to a higher survival rate by targeting cardiac iron metabolism [46].

### Antioxidant mechanisms

#### GPX4 dependent antioxidant mechanisms

The GPX family includes a number of isoenzymes that are expressed in various subcellular locations and organs. PL hydroperoxide can be reduced to PL alcohol by GPX4 [47, 48]. GPX4 primarily defends against ferroptosis by preventing the production of lipid peroxides in the cell membrane. Compared with solute carrier family 7 member 11 (SLC7A11) knockout mice, GPX4 knockout mice displayed early embryonic pathogenicity [49, 50]. This implies that GPX4 and SLC7A11 may have different functions in the synthesis of lipid peroxides. Selenium and glutathione (GSH) control GPX4 expression and function. GPX4 reduces cytotoxic lipid peroxide to the equivalent alcohol accompanied by oxidation of GSH. GSH is a cysteine-containing tripeptide that functions as an intracellular antioxidant, and its production is mainly dependent on the uptake of cystine and the conversion of cystine to cysteine, which is mediated by the amino acid reverse transport system SLC7A11 [18]. When SLC7A11 is suppressed by erastin, the transsulfuration pathway is upregulated, which could block ferroptosis [51]. GPX4 is inactivated immediately after GSH depletion. Glutamate cysteine ligase inhibitors, such as butylthiamine subfoximine, or system xc^−^ (xc^−^) cystine-glutamate antiporter inhibitors (erastin), can drive deficiencies of GSH [52]. The active domain of GPX4 is selenocysteine. The form of selenium used during GPX4 synthesis is selenocysteine, which is similar to cysteine but with sulphur substituted by selenium. Selenium can boost the anti-ferroptosis activity of GPX4 via selenocysteine residues at position-46 [53], and exogenous selenium supplementation inhibited ferroptosis in a mouse model of intracerebral haemorrhage [53–55].

When GPX4 activity is suppressed, lipid peroxides may build up and induce ferroptosis. Downregulated GPX4 expression increases the susceptibility of cells to ferroptosis, whereas upregulated GPX4 expression has the opposite impact [24]. Cystine starvation leads to GSH depletion and also induces glutamate accumulation, which inactivates GPX4 and ultimately leads to ferroptosis [52]. In addition, SLC7A11 activity is regulated in terms of protein–protein interactions, gene expression, and protein stability [56–60]. In addition to regulating GPX4 function, ferroptosis agonists also degrade GPX4 via autophagy or the ubiquitin–proteasome system [61–63]. Notably, GPX4 regulates cell apoptosis, necrosis, and pyrodeath in addition to ferroptosis [64–66].

#### GPX4-independent antioxidant mechanisms

Following the inactivation of GPX4, some cancer cells still remain resistant to ferroptosis [67, 68], implying that there are other ferroptosis defence mechanisms. Ferroptosis suppressor 1 (FSP1) inhibits ferroptosis independently of GPX4 [68, 69]. By reducing CoenzymeQ10 (CoQ), FSP1 prevents lipid peroxidation and suppresses ferroptosis [68, 69]. Similarly, in the mitochondrial inner membrane, dihydro-orotate dehydrogenase (DHODH) converts CoQ to CoQH2. By increasing DHODH activity when GPX4 is inactive, CoQH2 production is significantly increased and lipid peroxidation is neutralized, thereby mitigating ferroptosis in mitochondria [70]. The combination of GPX4 and DHODH in mitochondria inhibits mitochondrial lipid peroxidation. In addition to reducing CoQ, GTP cyclohydrolase-1 (GCH1) could produce tetrahydrobiopterin/dihydrobiopterin (BH4/BH2), thereby antagonizing ferroptosis independent of GPX4 by inhibiting lipid peroxidation [71].

## Ferroptosis and immune microenvironment

**Fig. 2:**
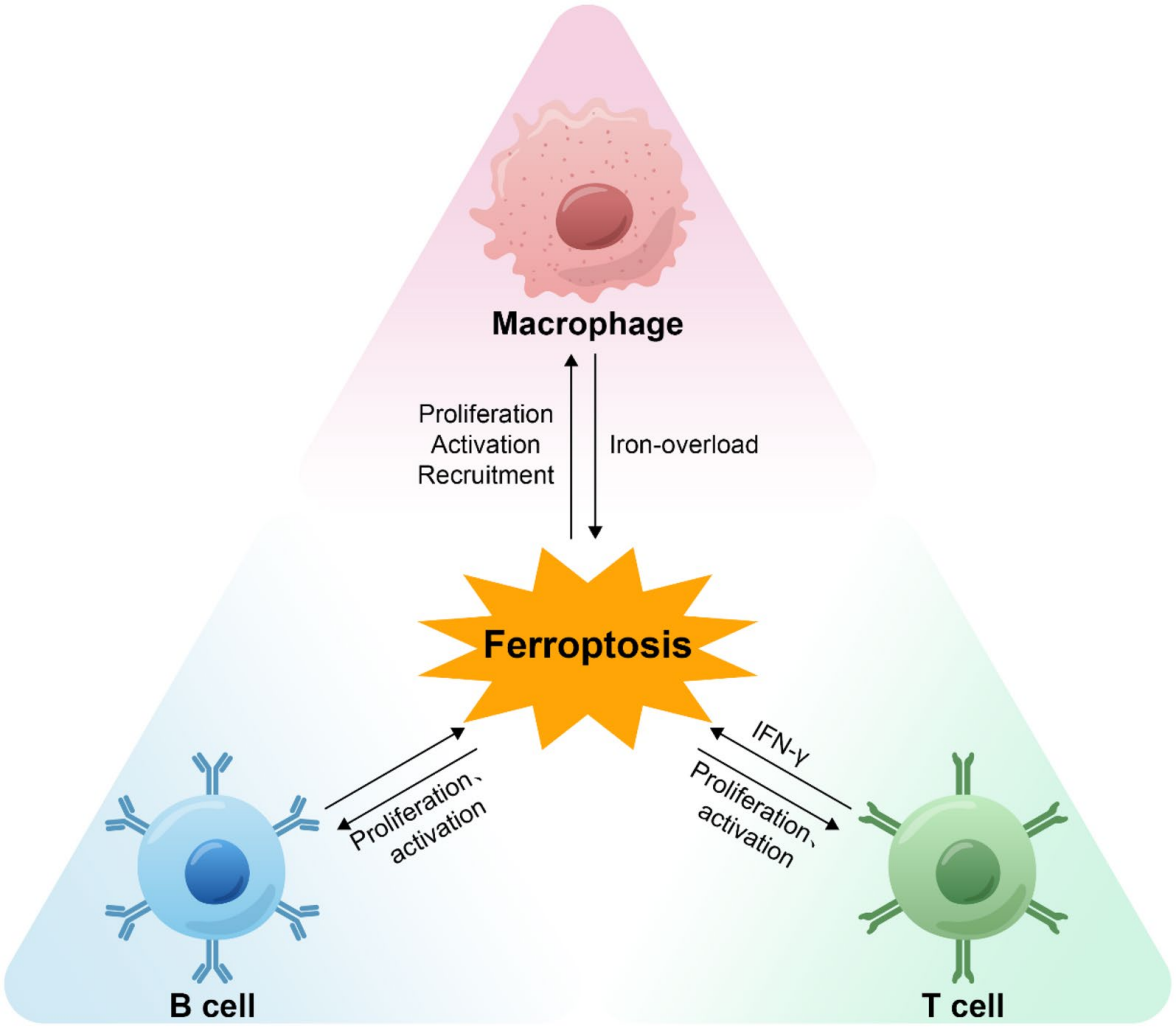
Interaction between ferroptosis and immune cells. Ferroptosis can promote the proliferation and activation of B cells, T cells, and macrophages. Ferroptosis also promotes the recruitment of macrophages. Macrophages induce ferroptosis by causing iron overload, and T cells induce ferroptosis by secreting IFN-γ

Regulated cell death is vital for maintaining homeostasis. In the immune system, ferroptosis is essential. The immune system is divided into two parts: inherent immunity and adaptive immunity. Ferroptosis can contribute to the immune process by influencing the quantity and function of immune cells. Additionally, immune cells can detect ferroptosis in non-immune cells and use it to trigger an immune response [72]. CVD is closely related to immune response [73–75]. The impact of ferroptosis on macrophages, T cells, and B cells will be covered in next section (Fig. 2).

### Ferroptosis and macrophages

Ferroptosis has a pro-inflammatory impact in macrophages [76] and this process can be blocked by RAS-selective lethal 3 [77]. GPX4 requirements in vivo are not consistent across macrophage subsets and are limited to alternatively activated macrophages whose proliferation or expansion is reduced after GPX4 deletion [78]. Macrophages usually store iron by binding to ferritin. At various stages of macrophage polarization, the expression of genes related to iron also varies. The expression of HAMP and ferritin L and H subunits was higher in M1 macrophages compared to M2 macrophages, while the expression of ferroportin 1 (FPN) and iron regulatory protein 1/2 was lower, suggesting a greater ability to store iron and a stronger resistance to ferroptosis [79, 80]. Iron overload promotes the polarization of M1 macrophages by increasing the levels of M1 markers interleukin-6, tumour necrosis factor, and interleukin-1 and decreasing the levels of M2 marker tissue transglutaminase-2 [81].

Similarly, the malondialdehyde content and Fe2+ load were substantially increased in mice with myocardial infarction, accompanied by ferroptosis of myocardial cells. In heart tissue from myocardial infarction mice, the expression of the M1 marker nitric oxide synthase 2 (NOS2) was substantially upregulated compared with that of the control group, while the expression of the M2 marker interleukin-10 was significantly downregulated [82]. In the context of polycythemia, macrophages can induce iron death by phagocytosis of erythrocytes, thus exacerbating AS [83].

### Ferroptosis and T cells

T cells are engaged in adaptive immunity, which includes responses to pathogens, allergens, and tumours [84]. T cell activity and function are regulated by ferroptosis. SLC7A11 is almost completely absent from human naive CD4+ T cells but is markedly increased during the T cell activation process [85]. T cell activation and proliferation require the maintenance of intracellular GSH levels [86]. T cells undergo ferroptosis when the GPX4 level is diminished or lipid peroxidation levels are elevated. T cell mortality induced by the glucocorticoid and diterpene compound Kayadiol entails ferroptosis [87, 88]. In contrast, loss of ACSL4 or overexpression of GPX4 can prevent ferroptosis in T cells [89–92]. Interestingly, ferroptosis is an important mechanism of T cell activation and immune function [92–94]. Vitamin E supplementation is a treatment option for GPX4 insufficiency because it prevents CD4+ or CD8+ T cells from expanding in the event of an acute infection [95].

The acute rejection of transplanted hearts has a negative impact on the therapeutic result. The number of CD3+, CD4+, and CD8+ cells in spleen cells and draining lymph node cells was substantially decreased in tumour necrosis factor-induced protein-8 −/− mice, and CD4+ and CD8+ cells also showed a decreased capacity to produce interferon based on activation of the TANK-binding kinase 1 signalling axis and the upregulation of GPX4. Interferon-γ promotes lipid peroxidation associated with ferroptosis in cardiomyocytes and is inhibited by GPX4 expression. Thus, heart allograft damage is significantly reduced by inhibiting ferroptosis [96]. Ferroptosis has great application potential in the treatment of a variety of diseases via its ability to influence immune response involving T cells.

### Ferroptosis and B cells

Hematopoietic stem cells (HSCs) are the source of B cells, which grow through a number of stages before maturing into B cells, including early lymphoid progenitors, common lymphoid progenitors, pre-B cells in bone marrow, and transitional B cells in peripheral lymphoid organs [97]. During B1 and marginal zone B cell development, maintenance, and immunity, GPX4 is necessary to promote ferroptosis. However, GPX4 plays the opposite role in follicular B2 cell development, germinal centre response, and antibody response, which is because B1 and marginal zone B cells are more susceptible to lipid peroxidation and ferroptosis than follicular B2 cells [98]. In chickens, melatonin causes a significant increase in malondialdehyde content and inducible nitric oxide synthase expression and a significant decrease in superoxide dismutase, GSH peroxidase, and total antioxidant capacity, which ultimately promotes B cell proliferation [99]. Interestingly, both inadequate and excessive GPX4 concentrations inhibited B cell proliferation [100]. The impact of GPX4 on B cell proliferation requires additional research. The ferroptosis agonist erastin induces lipid peroxidation that promotes the proliferation and differentiation of human peripheral blood mononuclear cells into B cells and natural killer cells by downregulating the expression of bone morphogenetic protein family members [101]. B cells produce antibodies that interfere with cardiomyocyte function, recruit a variety of immune cells, and play an important role in heart failure [102]. Interestingly, regulatory B cells reduce the expression of C–C motif chemokine receptor 2 (CCR2) in monocytes, thereby inhibiting the mobilization of pro-inflammatory monocytes and ultimately limiting ventricular remodelling after myocardial infarction [103].

## Ferroptosis and cardiovascular diseases

**Fig. 3:**
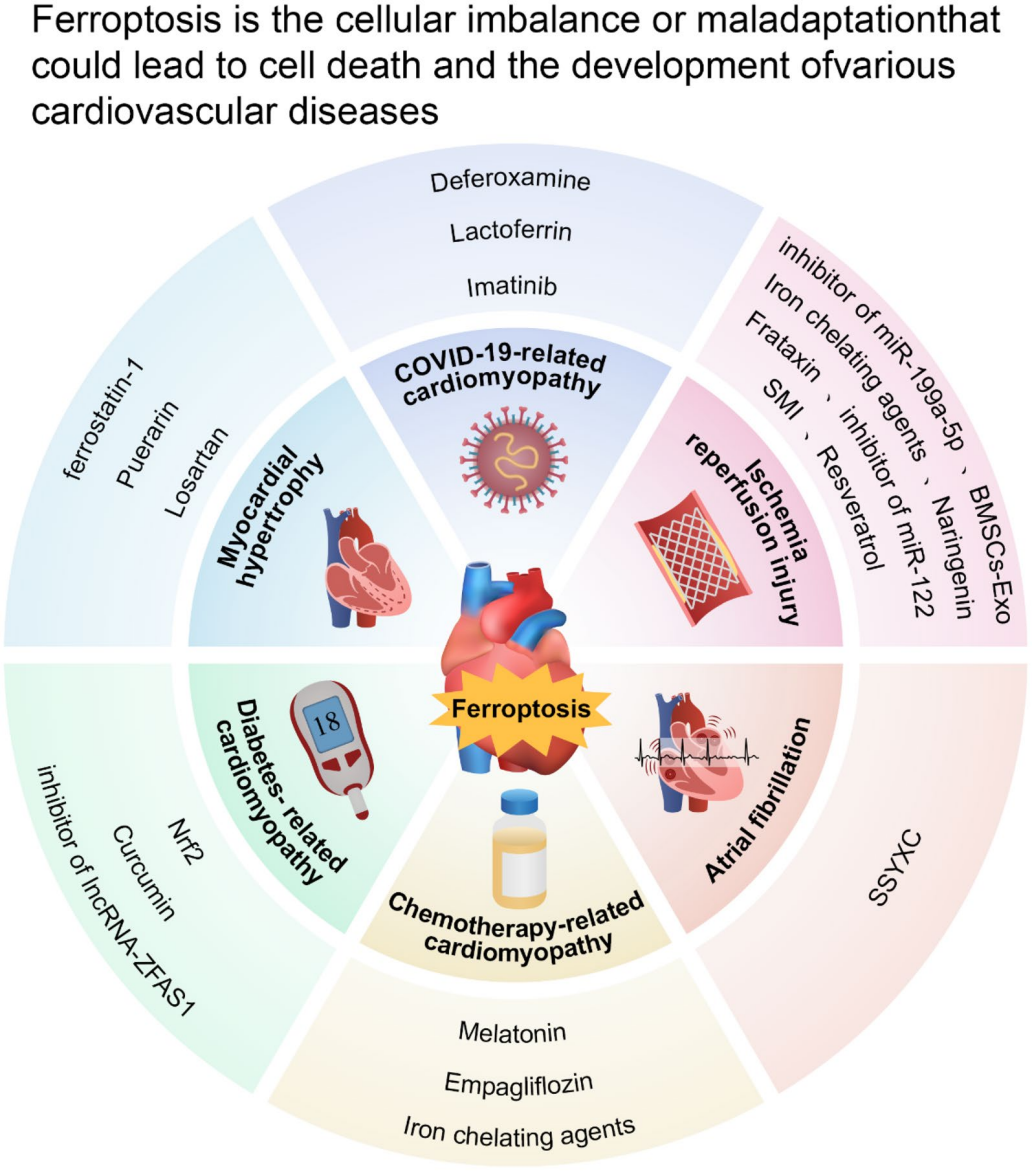
Ferroptosis-related cardiovascular diseases and potential treatment reagents. Ferroptosis is involved in the occurrence and progression of ischemia perfusion injury, atrial fibrillation, chemotherapy-related cardiomyopathy, diabetes-related cardiomyopathy, myocardial hypertrophy, and COVID-19-related cardiomyopathy. Molecules and drugs targeting ferroptosis may ameliorate these ferroptosis-related heart diseases, and they include SMI, SSYXC, melatonin, curcumin, ferrostatin-1, and deferoxamine

In this section, we summarize the relationship between ferroptosis and various CVDs. Patients with hereditary hemochromatosis often have iron overload, myocardial hypertrophy, and decreased left ventricular ejection fraction [104]. An increasing body of research has implicated ferroptosis in a number of CVDs, including myocardial I/R injury, cardiomyopathy, arrhythmia, chemotherapy, and heart injury caused by Corona Virus-2 (CoV-2) (Fig. 3).

### Ischemia reperfusion (I/R)

The leading cause of death and disability globally is acute myocardial infarction (MI). The best method of reducing acute myocardial ischemia injury and decreasing the size of myocardial infarction is through prompt and efficient myocardial reperfusion. However, I/R can cause myocardial reperfusion injury, which results in myocardial cell death [5]. Ferroptosis occurs in the early stages of I/R injury and represents the dominant form of cell death during prolonged reperfusion [105]. Ischemia triggers redox reaction between PUFAs and phospholipids in cardiomyocytes, which results in powerful oxidative damage during the reperfusion stage [106]. Therefore, ferroptosis plays an important role in I/R injury [107]. Iron chelating agents can protect cardiomyocytes from cardiac iron deposition during I/R [10]. Oxidation of phosphatidylcholine during reperfusion produces phosphatidylcholine oxide (OxPC), which disrupts mitochondrial biological function and calcium transport and induces ferroptosis, leading to widespread cell death. However, cell death during reperfusion is prevented by neutralizing OxPC. OxPC produced during reperfusion injury is a potent inducer of cardiomyocyte death [108]. In I/R, bone marrow stromal cell exo-culture demonstrated increased cell proliferation and GSH content and decreased iron concentration, ROS levels, and iron death marker protein levels. As a result, ferroptosis of cardiomyocytes was inhibited, I/R-induced cardiac injury was reduced, and cardiac function was enhanced [109]. Another study showed that the levels of the non-coding RNA miR-199a-5p were increased in I/R [110]. Cardiomyocyte mortality after ferroptosis can be improved by inhibitors of ferroptosis. MiR-199a-5p blocks the signalling cascade involving Akt serine/threonine kinase and endothelial nitric oxide synthase; thus, it is a key player in promoting iron damage-induced cardiomyocyte death during ischemia/hypoxia injury [110]. Similarly, blocking miR-122, which specifically targets GPX4, can produce protective effects against I/R injury in vitro [111]. Compared with animals lacking the cardiomyocyte-specific hypoxia-inducible factor-1 (HIF-1), control mice with I/R injury presented increased frataxin expression in their hearts. Frataxin can preserve mitochondrial membrane integrity and normal cardiomyocyte function by reducing ROS generation and iron overload in the mitochondria, and it may be an iron-storing protein [112].

Thus, the above findings indicate that ferroptosis is a potential therapeutic target for myocardial I/R injury. In I/R rats, resveratrol reduced oxidative stress levels and Fe2+ content and increased GPX4 expression, thereby inhibiting ferroptosis. Additionally, resveratrol prevents ferroptosis by controlling ubiquitin-specific peptidase 19-Beclin1 autophagy. Thus, resveratrol may be a potential drug for preventing myocardial I/R damage [113]. The nuclear factor erythroid 2-related factor 2 (NRF2)/System xc-/GPX4 pathway is regulated by naringenin, which can prevent ferroptosis, and erastin reversed the naringenin-mediated protective effects of cardiomyocytes. Therefore, maringenin regulates the Nrf2/System Xc-/GPX4 axis to suppress ferroptosis, which reduces myocardial I/R injury [114]. Shenmai injection can also reduce cardiac I/R injury by activating the Nrf2/GPX4 signalling pathway [115]. In addition, dapagliflozin can reduce MI-reperfusion injury via inhibiting ferroptosis by modulating MAPK signalling pathways [116].

### Atrial fibrillation

Atrial fibrillation (AF), a prevalent arrhythmia in clinical settings, is linked to high clinical mortality rates. Patients with AF usually die not from AF but from its concomitant complications, such as heart failure (HF), MI, venous thromboembolism (VTE), and stroke. According to data from the Framingham Heart Study, the incidence of AF has quadrupled over the past 50 years [117]. The occurrence of AF is related to many pathological factors; for example, frequent and excessive alcohol consumption can activate ferroptosis and increase the incidence of AF. However, ferroptosis inhibitors can reduce the susceptibility to AF [118]. Therefore, ferroptosis may contribute to the development of AF. Ferroptosis activation was later discovered to significantly increase the susceptibility to AF in rat models of endotoxemia, canine rapid atrial pacing, and chronic iron overload mice, whereas ferroptosis inhibition was found to reverse this change [119–121]. Due to cardiac iron deposition, beta-thalassemia patients have a higher risk factor for developing AF [122]. In these patients, iron chelators might help prevent AF. Taken together, ferroptosis inhibitors may be promising therapeutic targets for the prevention and treatment of AF in a range of pathological situations. Shensong Yangxin capsule can reduce the susceptibility of AF and inhibit electrical and structural remodelling in patients with metabolic syndrome by upregulating FPN and inhibiting ferroptosis. This indicates that the Shensong Yangxin capsule may be a potential drug to treat AF caused by metabolic syndrome [123].

### COVID-19-related cardiomyopathy

Coronavirus disease 2019 (COVID-19), which is caused by the severe acute respiratory syndrome coronavirus type 2 (SARS-CoV-2), sparked a global pandemic. Either the acute or late acute phase of COVID-19 may induce cardiovascular complications, such as palpitations, chest pain, stress cardiomyopathy, myocarditis, postural tachycardia, arrhythmias, and MI [124–129]. In patients with COVID-19, SARS-CoV-2 can inhibit the expression of a specific set of selenoprotein mRNAs and suppress GPX4 activity [130]. In addition, SARS-CoV-2 ORF3a promotes NRF2 degradation through the recruitment of Keap1, thus weakening the resistance of cells to oxidative stress and promoting ferroptosis [131]. In addition, coronavirus can induce ferroptosis via ACLS4. Interestingly, by inhibiting ACLS4, coronavirus replication is reduced [132]. Thus, ferroptosis has been suggested as a possible therapeutic target for the treatment of COVID-19. Following SARS-CoV-2 infection, sinoatrial node cells in the heart develop ferroptosis and exhibit dysfunction. Iron chelators deferoxamine and lactoferrin and the tyrosine kinase inhibitor imatinib prevent viral infection and ferroptosis-associated injury [133, 134].

### Diabetes-associated cardiomyopathy

Diabetes is very prevalent in patients with CVDs. Myocardial fibrosis, hypertrophy, and cardiac diastolic dysfunction are characteristics of diabetic cardiomyopathy, which is distinct from coronary artery disease and hypertension and eventually results in HF. Diabetic cardiomyopathy is primarily caused by insulin resistance, type 2 diabetes, and the resulting hyperinsulinemia [135]. The progression of the illness is mediated by a number of mechanisms, including oxidative stress [136]. In diabetic retinopathy, where autophagy serves a protective role, ferroptosis has been implicated in the death of pigment epithelial cells [137]. Similarly, ferroptosis inhibitors prevent high-glucose-induced cardiomyocyte dysfunction in diabetes-induced cardiomyopathy, suggesting that ferroptosis may play an essential part in the development of diabetic cardiomyopathy [12].

Ferroptosis was activated in a diabetic rabbit model. Curcumin can reduce cardiac injury caused by ferroptosis and enhance cardiac performance by increasing Nrf2 nuclear translocation and GPX4 and haem oxygenase-1 (HO-1) expression [138]. The activation of HO-1, a mitochondrial enzyme that catalyses the degradation of haem to generate ferrous iron, results in increased mitochondrial iron, thereby upregulating ferroptosis [139, 140]. Curcumin might also regulate ferroptosis by upregulating ferritin and SLC7A11 levels; moreover, NRF2 stimulation prevents ferroptosis [141]. Non-coding RNAs are also crucial in the development of diabetic cardiomyopathy. lncRNA-ZFAS1 can promote the progression of diabetic cardiomyopathy by downregulating cyclin-D2, thereby facilitating ferroptosis. These findings suggest a potential strategy for treating and preventing diabetic cardiomyopathy by targeting lncRNA-ZFAS1 [142].

Patients with diabetes are more likely to develop coronary heart disease. The optimum treatment for acute MI is prompt revascularization. Revascularization may lead to I/R injury, thereby impairing clinical benefit [143]. Activation of ferroptosis in diabetic patients increases the vulnerability of the heart after I/R. The NRF2/FPN1 signalling pathway can inhibit ferroptosis by regulating iron metabolism homeostasis, which partially alleviates myocardial reperfusion injury in diabetes mellitus [144]. One effective strategy for the prevention and management of diabetic myocardial I/R injury may be to inhibit ferroptosis by controlling iron metabolism.

### Chemotherapy-related cardiomyopathy

One of the major side effects of applying doxorubicin is potentially fatal cardiovascular toxicity, such as congestive HF and cardiomyopathy [145]. Doxorubicin can inhibit GPX4 and NRF2 production, thereby causing lipid peroxidation and ultimately ferroptosis. However, GPX4 upregulation or Fe2+ chelation in mitochondria prevents doxorubicin-induced mitochondrial ferroptosis [146, 147]. In addition, the FoxO4/ectonucleotide pyrophosphatase/phosphodiesterase family member 2 axis and the TRIM21/NRF2 axis can inhibit ferroptosis, thereby alleviating doxorubicin-induced cardiotoxicity. These findings suggest an effective treatment for doxorubicin cardiotoxicity [148, 149].

Melatonin and empagliflozin can inhibit ferroptosis and protect against doxorubicin-induced cardiotoxicity. Melatonin reverses the doxorubicin-induced upregulation of ACSL4 and downregulation of GPX4 and yes-associated protein 1 (YAP1). By participating in NLRP3 and NF-kB related signalling pathways, empagliflozin, a new hypoglycaemic drug that can improve the prognosis of CVDs, can attenuate ferroptosis, fibrosis, apoptosis, and inflammation in doxorubicin-treated mice and significantly improve cardiac function, thereby opening up a new avenue for the treatment of doxorubicin-related cardiotoxicity [150, 151]. Nevertheless, myocardial ferroptosis can be exacerbated by inducing a histamine deficit or pharmacologically inhibiting the histamine H1 receptor. Disruption of the histamine/histamine H1 receptor signalling axis regulates the signal transducer and activator of transcription 3 (STAT3)-SLC7A11 pathway, which may exacerbate doxorubicin-related cardiotoxicity [152]. This finding suggests possible adverse effects of antihistamines in patients treated with doxorubicin.

### Hypertrophic cardiomyopathy

In the heart, high blood pressure and aortic stenosis often lead to hypertrophic cardiomyopathy, fibrosis and eventually HF. Cardiac hypertrophy and decreased left ventricular ejection fraction have been observed in patients with hereditary hemochromatosis [104], among whom heart damage caused by iron overload is the leading cause of death. Age-related increases in cardiac iron levels were observed in a hemochromatosis mouse model, which suggests that iron deposition and elevated levels of oxidative stress occurred [153]. Thus, ferroptosis may be involved in heart hypertrophy in hemochromatosis patients.

A high-iron diet causes decreased GSH levels and increased lipid peroxidation, which lead to significant heart damage and hypertrophic cardiomyopathy. These changes were reversed by ferrostatin-1, thus providing solid evidence that ferroptosis contributes to ventricular hypertrophy [154]. Cardiomyocytes treated with angiotensin II underwent hypertrophy due to the decrease of xCT mRNA and protein levels. Knocking out xCT can exacerbate cardiac hypertrophy and dysfunction. Similarly, ferrostatin-1 and Elabela may reduce heart remodelling by inhibiting ferroptosis [155, 156]. Cardiac hypertrophy is associated with abnormal cardiac microvascular function [157]. SLC7A11 transcription is downregulated and ferroptosis is promoted when interferon regulatory factor 3 (IRF3) is inhibited. However, when docosahexaenoic acid is applied, IRF3 expression is increased and the endothelial system is protected from pressure overload. This finding implies that up-regulating IRF3 may offer possible therapeutic approaches for the management of HF and cardiac hypertrophy [158]. In human and mouse hypertrophic heart models and mice injected with apelin-13, cardiac mitochondrial iron deposition was significantly increased and NCOA4 and sideroflexin 1 (SFXN1) expression was elevated. Apelin-13-induced mitochondrial iron excess is reversed by inhibiting SFXN1 and NCOA4 expression, which it also mitigates cardiac hypertrophy. Furthermore, NCOA4 suppression prevented the rise in SFXN1 expression brought on by apelin-13. This indicates that NCOA4 functions upstream of SFXN1 and implicates NCOA4-mediated autophagy and ferroptosis in the development of cardiac hypertrophy [159, 160]. The expression of ATP-binding cassette subfamily B member 7 and mitochondrial oxidative phosphorylation enzymes were significantly downregulated in rats with left ventricular hypertrophy, while lipid metabolites, iron, ROS, and autophagy-associated proteins were upregulated in the cytoplasm and mitochondria. However, by interacting with mitochondrial complexes IV and V, ATP-binding cassette subfamily B member 7 reverses the above process when it is overexpressed [161].

Puerarin has been found to ameliorate HF in the clinic, and its anti-cardiomyocyte cell death function has been confirmed by animal studies. Puerarin can inhibit cardiac ferroptosis and protect cardiac function in HF mice with over-afterload. This suggests that puerarin may be a potential treatment strategy for HF [162]. Mice receiving intravenous iron supplementation showed increased iron deposition in lung tissue, increased pulmonary artery resistance, and right heart hypertrophy. Losartan, an angiotensin II-1 receptor blocker, prevents iron overload-induced vascular remodelling, pulmonary hypertension, and right ventricular hypertrophy [163].

## Ferroptosis-associated therapeutic opportunities

Ferroptosis is linked to the occurrence and progression of a number of heart illnesses, including myocardial I/R injury, AF, hypertensive heart disease, diabetic heart disease. There are novel opportunities for the treatment of these diseases based on the involvement of ferroptosis (Table 1). In myocardial I/R damage, iron-chelating agents can be protective [102]. Exosomes and non-coding RNA are also implicated in the induction of iron death, and their inhibitors could be used as potential therapeutic targets [109–111]. Resveratrol, naringenin and Shenmai injection have shown protective effects on reperfusion of myocardium [113–115]. The likelihood of AF can be decreased by iron-chelating medications [122], and it may also be reduced by Shensong Yangxin capsules by inhibiting the ferroptosis pathway [123]. Imatinib, the iron-chelating agent deferoxamine, and lactoferrin all showed protective effects in ferroptosis-induced cardiac dysfunction induced by COVID-19 [133, 134]. In diabetic cardiomyopathy, non-coding RNA may be involved in disease progression by inducing ferroptosis. Therefore, specific non-coding RNA inhibitors can play a protective role [142]. In addition, curcumin has been shown to inhibit ferroptosis and play a cardioprotective role [138]. Doxorubicin treatment-related heart damage is a long-term clinical concern, although studies have found that melatonin and empagliflozin may have a heart-protective effect by inhibiting ferroptosis. In addition, empagliflozin is frequently used to manage diabetes [150, 151]. Losartan and puerarin can inhibit ventricular hypertrophy by inhibiting ferroptosis. Losartan has been used in the clinic, and puerarin is a potential therapeutic target [162, 163].

**Table 1:** Cardiovascular disease treatment strategies involving ferroptosis

Reagents	Mechanisms	CADs	References
BMSCS-EXO	Increases GSH level	Myocardial I/R injury	[88]
FRATAXIN	Reduces mitochondrial iron and ROS level	Myocardial I/R injury	[91]
RESVERATROL	Regulates USP19-Beclin1 autophagy	Myocardial I/R injury	[92]
NARINGIN	Regulates the NRF2 /System xc—/GPX4 pathway	Myocardial I/R injury	[93]
SMI	Activates Nrf2/GPX4 pathway	Myocardial I/R injury	[94]
SSYXC	Increases transferrin level	Atrial fibrillation	[164]
DESFERRIAMINE	Inhibits iron overload	COVID-19-associated cardiomyopathy	[104]
LACTOFERRIN	Inhibits iron overload	COVID-19-associated cardiomyopathy	[107]
MELATONIN	Reduces ACSL4 level and increases GPX4 level	Chemotherapy-related cardiomyopathy	[121]
EMPAGLIFLOZIN	Regulates NLRP3 and myd88 related pathways	Chemotherapy-related cardiomyopathy	[122]
FERROSTATIN-1	Inhibits lipids peroxidation	Hypertrophic cardiomyopathy	[127]
DHA	Increases SLC7A11 level	Hypertrophic cardiomyopathy	[165]
ABCB7	Interacts with mitochondrial complex IV and V	Hypertrophic cardiomyopathy	[134]
PUERARIN	Inhibits iron overload and lipids peroxidation	Hypertrophic cardiomyopathy	[135]
Losartan	Inhibits iron overload	Hypertrophic cardiomyopathy	[136]

In ferroptosis-related heart disease, a variety of effectors have been identified that play a cardioprotective role either by inhibiting oxidation mechanisms or by regulating antioxidant mechanisms, including the ferroptosis pathway. However, further research is needed before these agents can be used in the clinic.

## Conclusions and perspectives

One of the main threats to human life and health is CVD. To improve patient quality of life and save lives, advances in the treatment of CVD are desperately needed. Ferroptosis has recently been identified as a regulated mode of cell death, and a growing number of studies have demonstrated that ferroptosis is closely related to the occurrence and development of various CVDs. Studies have revealed the role of ferroptosis in heart disease and its regulatory mechanisms and thus have provided mechanistic insights into CVDs and novel treatment options. However, the role of ferroptosis in this field is not sufficiently understood to develop an efficient treatment strategy.

The field of ferroptosis is being actively studied. More research is needed to refine our understanding of the regulatory mechanisms of ferroptosis. In addition, the inducers and inhibitors of ferroptosis need to be further clarified so that more substances with stronger application potential can be screened to serve as treatments. There are many modes of regulated cell death, and they are not completely independent of each other. Thus, further research is needed to determine the relationship between different regulated cell death modes and reveal how they interact with each other. Such work would provide a deeper understanding of the occurrence and development of living processes and may reveal better disease treatment strategies.

## Data Availability

Not applicable.

## Abbreviations

CVD: Cardiovascular disease
ROS: Reactive oxygen species
PUFAs: Polyunsaturated fatty acids
GPX4: Glutathione peroxidase 4
AS: Atherosclerosis
ACSL4: Long-chain-fatty-acid-CoA ligase 4
LPCAT3: Lysophosphatidylcholine acyltransferase 3
AdA: Adrenic acid
MUFAs: Monounsaturated fatty acyl tails
NCOA4: Nuclear Receptor Coactivator 4
SLC7A11: Solute carrier family 7 member 11
GSH: Glutathione
FSP1: Ferroptosis suppressor 1
NOS2: Nitric oxide synthase 2
HSCs: Hematopoietic stem cells
MI: Myocardial infarction
HIF-1: Hypoxia-inducible factor-1
NRF2: Nuclear factor erythroid 2-related factor 2
AF: Atrial fibrillation
HF: Heart failure
VTE: Venous thromboembolism
COVID-19: Coronavirus disease 2019
SARS-CoV-2: Severe acute respiratory syndrome coronavirus type 2
HO-1: Haem oxygenase-1
YAP1: Yes-associated protein 1
STAT3: Signal transducer and activator of transcription 3
IRF3: Interferon regulatory factor 3
SFXN1: Sideroflexin 1
